# Operative management of a non-traumatic cervico-thoracic spondylolisthesis: a case report

**DOI:** 10.1186/1752-1947-6-146

**Published:** 2012-06-12

**Authors:** Stefan Zwingenberger, Mario Leimert, Roberto D Valladares, Volker M Betz, Jens Seifert

**Affiliations:** 1 Department of Orthopaedics, University Hospital Carl Gustav Carus at Technical University, Fetscherstraße 74, Dresden, D-01307, Germany; 2 Department of Neurosurgery, University Hospital Carl Gustav Carus at Technical University, Fetscherstraße 74, Dresden, D-01307, Germany; 3 Department of Orthopaedic Surgery, Stanford University Medical Center Outpatient Center, 450 Broadway Street M/C 6342, Redwood City, CA, 94063, USA; 4 Department of Trauma & Reconstructive Surgery, University Hospital Carl Gustav Carus at Technical University, Fetscherstraße 74, D-01307, Dresden, Germany

**Keywords:** Cervico-thoracic, Spondylolisthesis, Myelopathy, Screws, Transverse processes

## Abstract

**Introduction:**

In contrast to spondylolisthesis of the lumbar spine, non-traumatic cervico-thoracic spondylolisthesis is a very rare lesion. Even minor changes in the displacement of the vertebrae or the cord can lead to cervical myelopathy and paralysis. Since only a few cases have been well-documented, there is currently no clear preference between operative techniques.

**Case presentation:**

We describe the case of a 63-year-old Caucasian man with a 13 mm spondylolisthesis between C7 and T1. Within a few months, a progressive cervical myelopathy developed as he began to suffer pain and loss of function of his digits and was no longer able to walk unassisted. In an interdisciplinary collaboration between neurological and orthopedic surgeons, a ventral-dorsal-ventral approach was performed on one vertebral section. The ventral removal of the intervertebral disc was followed by laminectomy and dorsal instrumentation. A new application technique was established by inserting bicortical screws into the transverse processes of T2 and T3. The structure was subsequently stabilized by the ventral insertion of a Harms basket. The procedure was successful as it halted progression of the myelopathy. The patient demonstrated improved sensitivity and recovered the ability to walk unassisted. He has now been able to walk unassisted for two years postoperatively.

**Conclusion:**

This paper describes a successful treatment for a very rare case of cervico-thoracic spondylolisthesis. The technique of inserting bicortical screws into the transverse processes is a fast, safe and successful method that does not require the use of intraoperative radiographs for placement of the bicortical screws into the transverse processes.

## Introduction

Lower cervical or cervico-thoracic spondylolisthesis may be caused by trauma, spondylodiscitis or degeneration of the facet joints, resulting in altered cervical mechanics and secondary subluxation [1-3]. Congenital lower cervical or cervico-thoracic spondylolisthesis is usually associated with a bilateral spondylolysis [4]. The most important mechanism that causes cervical spondylotic myelopathy is compression of the spinal cord [5].

While spondylolisthesis of the lumbar spine is a common disease, only a few cases of cervical spondylolisthesis have been well-documented. Compression of the lumbar spine results in a milder neurologic impairment than compression of the cervical spine, mainly due to the spinal cord ending around the level of L1. Even minor anatomical changes in the cervical region can lead to cervical myelopathy and paralysis.

Different scores are used for the evaluation of myelopathy. Although the Nurick Score (Table 1) [5], established in 1972, is one of the most popular scores, the gradation between its levels is very imprecise. The European Myelopathy Score (EMS, Table 2) [6] of 2008 presents a new and more detailed scoring system. In addition, we used Janda’s classification (J) [7] for grading muscular strength. This classification has six levels, ranging from zero for no muscular movement to five for full muscular strength.

**Table 1:** **Nurick classification system of myelopathy**[5]

Grade	Description of neurologic status
0	Signs or symptoms of root involvement but without evidence of spinal cord disease
1	Signs of spinal cord disease but no difficulty in walking
2	Slight difficulty in walking which did not prevent full-time employment
3	Difficulty in walking which prevented full-time employment or the ability to do all housework, but which was not so serve as to require someone else’s help to walk
4	Able to walk only with someone else’s help or with the aid of a frame
5	Chair bound or bedridden

**Table 2:** **European myelopathy score**[6]

Criterion	Clinical situation	Points
Upper motor neuron:gait function	Unable to walk, wheelchair	1
	Walking on a flat ground only withcane or aid	2
	Climbing stairs only with aid	3
	Gait clumsy, but no aid necessary	4
	Normal walking and climbing stairs	5
		
Upper motor neuron:bladder and bowel function	Retention, no control over bladder and/or bowel function	1
	Inadequate miction and urinary frequency	2
	Normal bladder and bowel function	3
		
Lower motor neuron:hand function	Handwriting and eating with knife and fork impossible	1
	Handwriting and eating with knife and fork impaired	2
	Handwriting, tying shoe laces, or a tie clumsy	3
	Normal handwriting	4
		
Posterior column: proprioception and coordination	Getting dressed only with aid	1
	Getting dressed clumsily and slowly	2
	Getting dressed normally	3
		
Paresthesia/pain	Invalidity because of pain	1
	Endurable paresthesia and pain	2
	No paresthesia and pain	3
		
Total score		5-18

The lower the score, the more severe the deficits. Normal function 17 to 18; Grade 1: 13 to 16; Grade 2: 9 to 12; Grade 3: 5 to 8.

## Case presentation

The patient is a 63-year-old Caucasian man. He is 1.61 meters tall and weighs 66 kilograms. After working as a car mechanic for 36 years, he had been receiving disability benefits for 11 years. He was working part-time as a caretaker averaging 10 hours per week until two and a half years ago. At the age of 35, the patient began having bouts of severe back pain approximately twice a year. When these episodes occurred, he took non-steroidal anti-inflammatory drugs for three to four weeks for pain relief. At the age of 40, lumbar spondylolisthesis was diagnosed by radiography. At the age of 50, he suddenly developed severe right hip pain. He suffered from substantial arthrosis on the right side, which was treated with a total hip replacement one year later.

**Figure 1:**
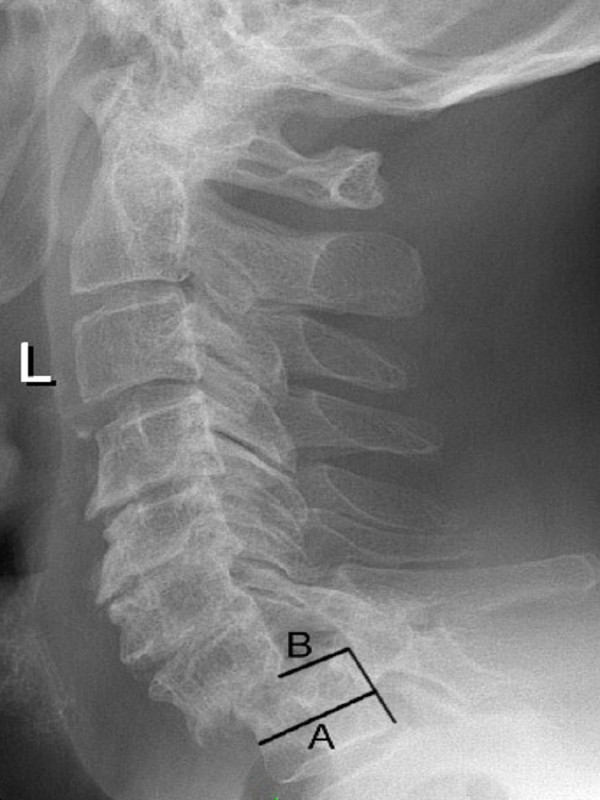
Lateral radiograph of the cervical spine shows a spondylolisthesis at C7-T1 (preoperative); the sagittal diameter of T1 (A) was 22.6 mm and the horizontal displacement (B) between C7 and T1 was 13 mm.

**Figure 2:**
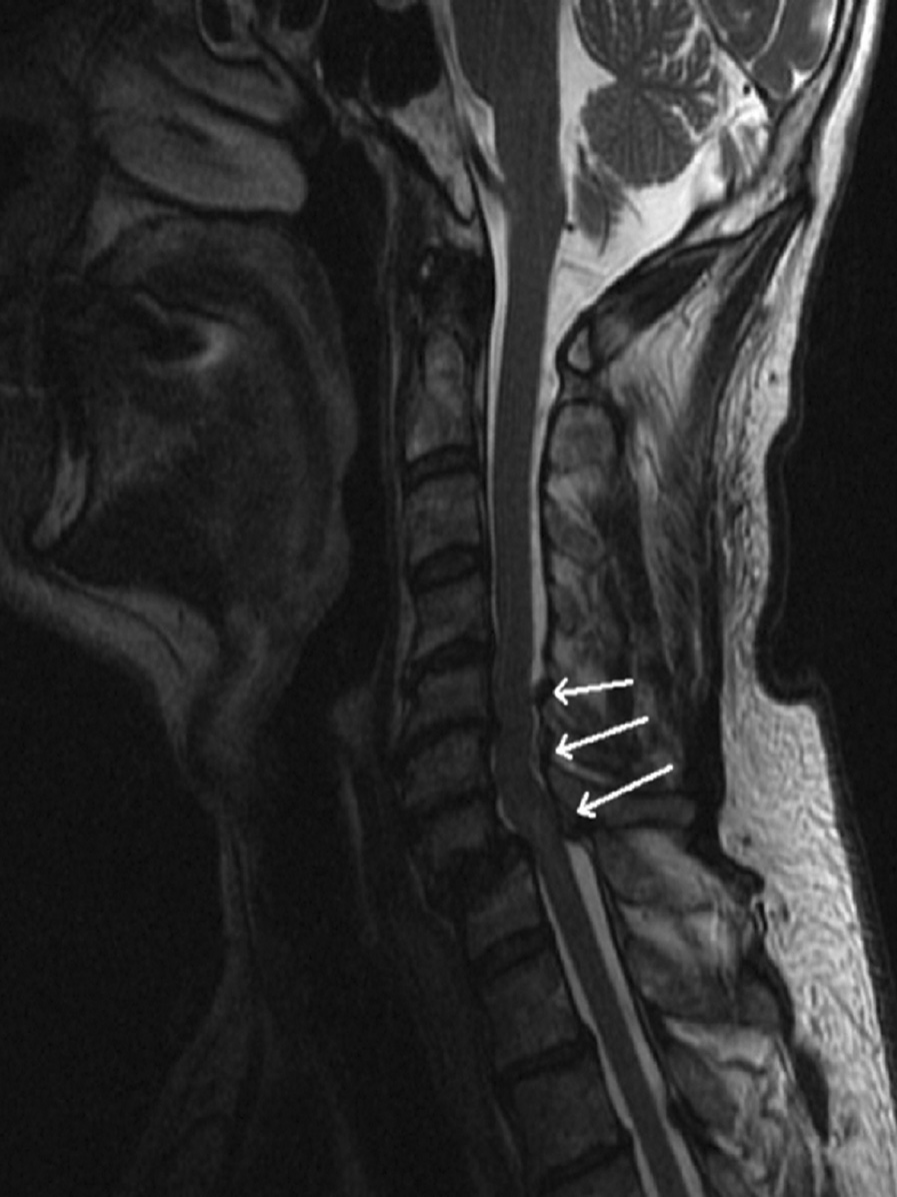
Magnetic resonance imaging of the cervical spine shows a compression of the spinal cord at C5-C6, C6-C7 und C7-T1 (preoperative).

Three years ago, he began to develop neurological symptoms in both hands. Pain and loss of function of his fingers prevented him from working as a caretaker. The pain radiated from his neck bilaterally down to his fingers and was described as parasthesia-like in nature. He also complained of weakness in his hands. Left hand digit flexion was classified as J1 and right hand as J4. Digit extension was classified as J4 on the left and J2 on the right and abduction on the left was classified as J0 and on the right as J1. The patient also had difficulty walking (Nurick 4, EMS 10/18) as he had to support himself using his surroundings in order to stand upright and was unable to walk unassisted. He was referred to a neurologist by his general practitioner who in turn referred him to a neurosurgeon. Radiographs (Figure 1) magnetic resonance imaging (Figure 2) and computed tomography (CT) revealed a spondylolisthesis between C7 and T1. Using the Meyerding classification [8], which was initially developed for grading the degree of lumbar spondylolisthesis, the patient would have been diagnosed with a cervico-thoracic spondylolisthesis of the second or third degree. The spondylolisthesis, measured using the method developed by Kawasaki *et al*. [9], was 13 mm. In view of the special nature of the case, a collaborative treatment between neurosurgeons and orthopedic surgeons was favored and subsequently implemented.

The surgical management was performed as follows:

The patient was placed in the supine position. The intervertebral disk at C7-T1 was exposed using blunt dissection. The disk was then removed to the point of the ligamentum flavum. A subsequent resection of the lateral parts of the intervertebral discs significantly mobilized the spondylolisthesis.

The wound was closed and the patient was rotated to the prone position. First, the dislocation between vertebras C7 and T1 was reduced while positioning the head.

Subsequently, the lateral masses were prepared from a dorsal approach between C5 and T3. Then, lateral mass-screws were inserted into C5 and C6. We had noticed considerable laxity between C6 and C7. In addition, C5 and C6 were naturally fused and we decided to include them in the instrumentation. There was also a rigid displacement between C7 and T1.

Bicortical screws were then inserted into the transverse processes of T2 and T3. These were inserted into at least two segments from both sides in divergent directions. The length of these screws was 10 to 12 mm, with a diameter of 3.2 mm.

This was followed by a laminectomy. Since the roots of C7 and C8 were exposed, the lateral masses of C7 and C8 had to be resected. Longitudinal rods were placed. Proper reduction in lordosis and the preservation of a 5 mm intervertebral space between C7 and T1 were confirmed by radiograph. Set screws were used to fix the instrumentation in the desired position. Chips of cortical and cancellous bone were placed lateral to the longitudinal rods.

The dorsal wound was then closed. The patient was again rotated to the supine position to reopen the ventral wound. Now, instead of the previously seen displacement, a large gap was visible between C7 and T1. The endplates between C7 and T1 were milled and the ligamentum flavum was resected using punches. Then, the spinal canal was exposed completely, using a Caspar opener.

**Figure 3:**
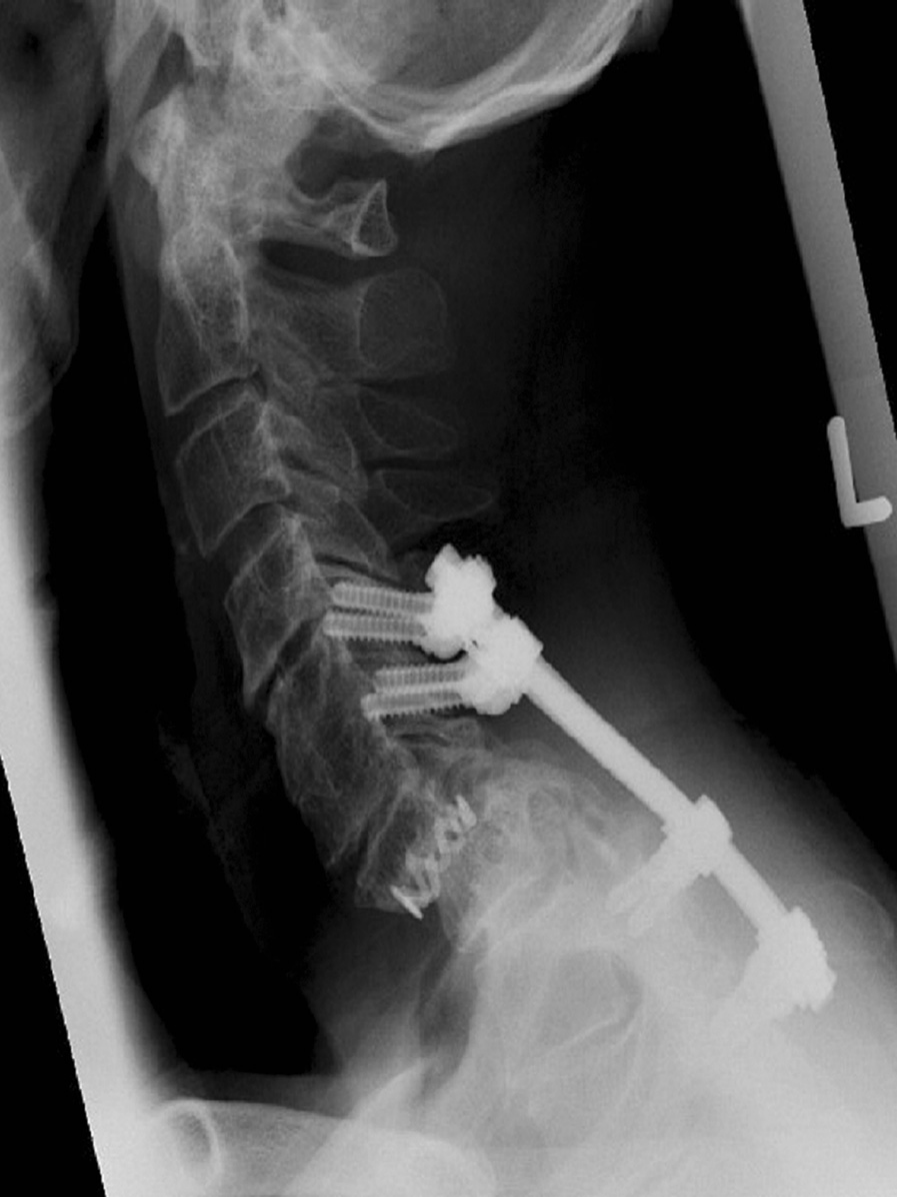
Lateral radiograph of the cervical spine shows the treatment of the spondylolisthesis at C7-T1.

**Figure 4:**
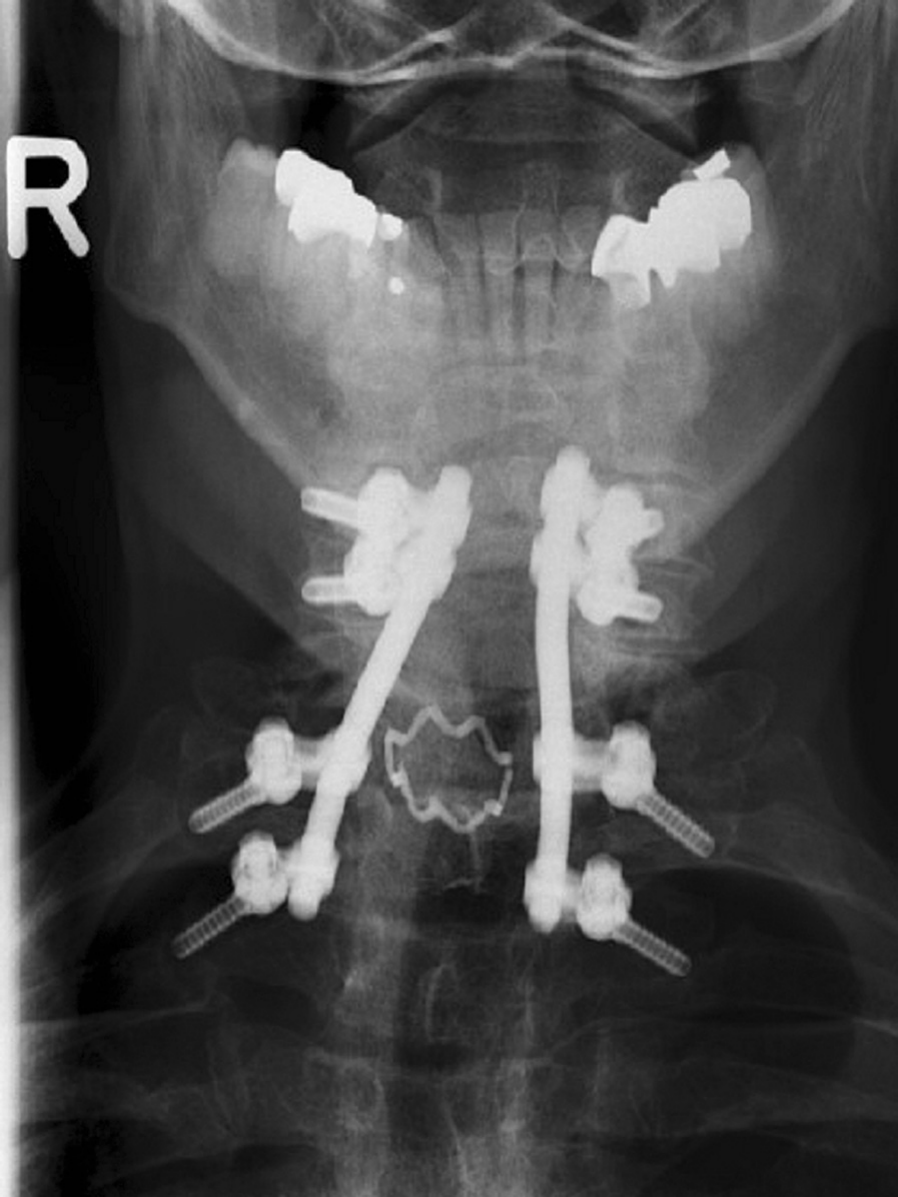
Anterior-posterior radiograph of the cervical spine shows the treatment of the spondylolisthesis at C7-T1; the lower screws were fixed bicortically into the transverse processes of T2 and T3.

Subsequently, a Harms basket, filled with autograft bone chips, was inserted into the intervertebral space. Three drains were placed and the wound was closed. For prophylactic infection control, the patient was perioperatively given intravenous 1.5 g cefuroxime twice a day for the next four days. Intraoperative and postoperative radiographs (Figure 3 and 4) were taken to confirm correct placement of the instrumentation.

Following the operation, the patient was transferred to the intensive care unit. His vital signs were stable enough for him to be transferred to the general postoperative recovery ward on the fourth postoperative day. Eleven days after surgery, the patient left the hospital and entered an orthopedic and neurologic rehabilitation program.

The pain and parasthesias in his fingers resolved after the operation. Two months postoperatively, his ability to walk had noticeably improved after completing his rehabilitation program (Nurick 3).

Today, two years postoperatively, the patient is able to walk without assistance (Nurick 2). Flexion of the fingers on his left hand was graded as J3 and as J5 on the right hand. Extension of the fingers on his left hand was graded as J4 and as J2 on the right. Abduction was graded as J4 for his left hand and as J2 for his right hand (EMS 14/18).

## Discussion

Decompression is the gold-standard to halt progression of cervical myelopathy caused by compression of the spinal cord. It is well-known that recovery of motor and sensory functions can be significantly delayed or may not occur at all.

Laminectomy is a conventional surgical treatment for cervical myelopathy [10]. It is a quick and easy procedure with minimal blood loss. Its significant disadvantages, however, include the loss of cervical lordosis, a decrease in the range of motion and the possible occurrence of neck and shoulder complaints [11]. It has been shown that compression of the spinal cord occurs in the articular segment rather than in the intraosseus segment. A segmental partial laminectomy may, therefore, be sufficient to reduce the loss of cervical lordosis [12]. A posterior stabilization at the time of the original decompressive procedure [13] may present another way to prevent the development of a kyphotic deformity. For the treatment of spondylolisthesis, posterior stabilization alone does not provide adequate stabilization. An anterior fusion, using a Harms basket filled with autograft bone, provides the highest degree of stabilization [14]. Posterior stabilization was necessary as a laminectomy and resection of the lateral masses of C7 and C8 was determined to be the safest method to decompress the spinal cord and the C7 and C8 nerve roots. A Harms basket was placed ventrally to increase the stability of the fusion and to avoid fatigue of the posterior stabilization.

In the case presented here, a ventral-dorsal-ventral approach was used. Using this technique, it is possible to visually inspect the spinal cord and the spinal nerves. Because there was a natural fusion of the vertebrae along C5 and C6 and because of the laxity between C6 and C7, it was not possible to use pedicle screws. Therefore, lateral mass screws had to be used. Irrespective of the case presented here, it has been shown that pedicle screws provide higher stability for lateral bending, flexion, extension and axial rotation compared to lateral mass screws [15]. The fact that placing the lateral mass screws is faster proved to be an advantage in this case, as it made it possible to perform the ventral-dorsal-ventral operation in a single surgical session.

A point of criticism in the case presented here may be the poor reduction of the spondylolisthesis. Multilevel cervical corpectomy [10], an alternative and popular surgical procedure, may present better options for an anatomic reduction in the treatment of degenerative cervical myelopathy. However, because of the high level of displacement of the vertebral bodies there was the danger of creating a spinal cord lesion during the reduction procedure. Consequently, the decision was taken to forego cervical corpectomy.

This new technique of placing bicortical screws into the transverse processes was successful in our case. In several complicated cases, such as tumor situations or cases of paraplegia of the upper thoracic spine, the senior author used this new technique of fixating small, bicortical screws into the transverse processes*.* This technique provides a fast and safe method that does not require the use of intraoperative radiography for placement of the bicortical screws into the transverse processes.

## Conclusion

No established surgical treatment exists for the case of a patient suffering from degenerative cervico-thoracic spondylolisthesis along with cervical myelopathy and a large displacement of the vertebrae. As this case involved aspects of the fields of neurosurgery and orthopedics, it was an advantage to implement an interdisciplinary treatment. A special technique was applied to place screws into the transverse processes. This innovative treatment succeeded in halting the rapid progression of this patient’s cervical myelopathy and he had a positive outcome.

## Consent

Written informed consent was obtained from the patient for publication of this case report and any accompanying images. A copy of the written consent is available for review by the Editor-in-chief of this journal.

## Competing interests

The authors declare that they have no competing interests.

## Authors’ contributions

SZ, ML and JS performed clinical work; the operation was done by ML and JS. All authors contributed to writing the article. All authors have read and approved the final manuscript.
